# Supplementary material for: Factors Associated with the Quality and Transparency of National Guidelines: A Mixed-Methods Study
Source: Int J Environ Res Public Health. 2022 Aug 3;19(15):9515. doi: 10.3390/ijerph19159515 (PMC9367745; doi:10.3390/ijerph19159515)
Supplement: Supplementary file 1 [file ijerph-19-09515-s001.zip › ijerph-1817884-supplementary.pdf]

Supplementary File

A more detailed explanation of the methodology used in quantitative and qualitative research

Content:

1. Methods – Quantitative study

2. Methods – Qualitative study

3. Supplementary figures and tables

4. References

1. Methods – Quantitative study

The usage of the AGREE II tool

As recommended by the developers of AGREE II, 4 raters were included in the study. They had different expertise: a research methodologist and evidence-based specialist, an emergency medicine physician, a general practitioner, and a paediatrician. The raters familiarised themselves with the AGREE II tool by reviewing PDF copies of the AGREE II user manual [1]. We did not translate the original AGREE II instrument from English to Croatian, since all evaluators had an excellent command of English. As additional training, each rater independently rated an international guideline translated to Croatian and discrepancies were discussed and resolved by consensus [2].

Each rater independently rated all 74 guidelines. The data analyst calculated the total score according to the instructions for the AGREE II tool and performed the statistical analysis.

Comparison with other countries

We only included studies that: a) used a representative sample of CPGs to assess the quality of national guidelines, b) estimated this quality with standardized domain scores of the AGREE II instrument, and c) reported the scores for all domains. This means that studies that examined national CPGs for a specific subset of diseases or that gathered CPGs published within just one year were excluded. Furthermore, the publication period of the CPGs examined in a study had to include more recent years; those for which this period overlapped with the last ten years (included year 2012 or later) were included in the comparison.

On July 25, 2022, we searched the MEDLINE database using the search filter below via the PubMed interface. We obtained a total of 209 records, and identified eight studies after applying inclusion and exclusion criteria. After reading the full texts of the collected studies, one study from Japan (Kataoka Y et al., 2021) was subsequently excluded because it examined only a subset of guidelines based on

systematic reviews, leaving a total of 7 studies (see legend to Figure 2 for references) from 6 countries: China, Japan, Chile, the Philippines, Peru, and Mexico; in the final data set.

PubMed was searched using the following search filter:
("guidelines as topic"[MeSH Major Topic]) AND (countr*[Title/Abstract] OR region[Title/Abstract] OR Afghanistan[Title/Abstract] OR Albania[Title/Abstract] OR Algeria[Title/Abstract] OR Andorra[Title/Abstract] OR Angola[Title/Abstract] OR "Antigua and Barbuda"[Title/Abstract] OR Argentina[Title/Abstract] OR Armenia[Title/Abstract] OR Australia[Title/Abstract] OR Austria[Title/Abstract] OR Azerbaijan[Title/Abstract] OR Bahamas[Title/Abstract] OR Bahrain[Title/Abstract] OR Bangladesh[Title/Abstract] OR Barbados[Title/Abstract] OR Belarus[Title/Abstract] OR Belgium[Title/Abstract] OR Belize[Title/Abstract] OR Benin[Title/Abstract] OR Bhutan[Title/Abstract] OR Bolivia[Title/Abstract] OR "Bosnia and Herzegovina"[Title/Abstract] OR Botswana[Title/Abstract] OR Brazil[Title/Abstract] OR Brunei[Title/Abstract] OR Bulgaria[Title/Abstract] OR "Burkina Faso"[Title/Abstract] OR Burundi[Title/Abstract] OR "Côte d'Ivoire"[Title/Abstract] OR "Cabo Verde"[Title/Abstract] OR Cambodia[Title/Abstract] OR Cameroon[Title/Abstract] OR Canada[Title/Abstract] OR "Central African Republic"[Title/Abstract] OR Chad[Title/Abstract] OR Chile[Title/Abstract] OR China[Title/Abstract] OR Colombia[Title/Abstract] OR Comoros[Title/Abstract] OR Congo[Title/Abstract] OR Costa Rica[Title/Abstract] OR Croatia[Title/Abstract] OR Cuba[Title/Abstract] OR Cyprus[Title/Abstract] OR Czech*[Title/Abstract] OR Congo[Title/Abstract] OR Denmark[Title/Abstract] OR Djibouti[Title/Abstract] OR Dominica[Title/Abstract] OR "Dominican*"[Title/Abstract] OR Ecuador[Title/Abstract] OR Egypt[Title/Abstract] OR Salvador[Title/Abstract] OR "Equatorial Guinea"[Title/Abstract] OR Eritrea[Title/Abstract] OR Estonia[Title/Abstract] OR Eswatini[Title/Abstract] OR Ethiopia[Title/Abstract] OR Fiji[Title/Abstract] OR Finland[Title/Abstract] OR France[Title/Abstract] OR Gabon[Title/Abstract] OR Gambia[Title/Abstract] OR Georgia[Title/Abstract] OR Germany[Title/Abstract] OR Ghana[Title/Abstract] OR Greece[Title/Abstract] OR Grenada[Title/Abstract] OR Guatemala[Title/Abstract] OR Guinea[Title/Abstract] OR Guinea-Bissau[Title/Abstract] OR Guyana[Title/Abstract] OR Haiti[Title/Abstract] OR "Holy See"[Title/Abstract] OR Honduras[Title/Abstract] OR Hungary[Title/Abstract] OR Iceland[Title/Abstract] OR India[Title/Abstract] OR Indonesia[Title/Abstract] OR Iran[Title/Abstract] OR Iraq[Title/Abstract] OR Ireland[Title/Abstract] OR Israel[Title/Abstract] OR Italy[Title/Abstract] OR Jamaica[Title/Abstract] OR Japan[Title/Abstract] OR Jordan[Title/Abstract] OR Kazakhstan[Title/Abstract] OR Kenya[Title/Abstract] OR Kiribati[Title/Abstract] OR Kuwait[Title/Abstract] OR Kyrgyzstan[Title/Abstract] OR Laos[Title/Abstract] OR Latvia[Title/Abstract] OR Lebanon[Title/Abstract] OR Lesotho[Title/Abstract] OR

Liberia[Title/Abstract] OR Libya[Title/Abstract] OR Liechtenstein[Title/Abstract] OR Lithuania[Title/Abstract] OR Luxembourg[Title/Abstract] OR Madagascar[Title/Abstract] OR Malawi[Title/Abstract] OR Malaysia[Title/Abstract] OR Maldives[Title/Abstract] OR Mali[Title/Abstract] OR Malta[Title/Abstract] OR "Marshall Islands"[Title/Abstract] OR Mauritania[Title/Abstract] OR Mauritius[Title/Abstract] OR Mexico[Title/Abstract] OR Micronesia[Title/Abstract] OR Moldova[Title/Abstract] OR Monaco[Title/Abstract] OR Mongolia[Title/Abstract] OR Montenegro[Title/Abstract] OR Morocco[Title/Abstract] OR Mozambique[Title/Abstract] OR Myanmar[Title/Abstract] OR Namibia[Title/Abstract] OR Nauru[Title/Abstract] OR Nepal[Title/Abstract] OR Netherlands[Title/Abstract] OR "New Zealand"[Title/Abstract] OR Nicaragua[Title/Abstract] OR Niger[Title/Abstract] OR Nigeria[Title/Abstract] OR "North Korea"[Title/Abstract] OR "North Macedonia"[Title/Abstract] OR Norway[Title/Abstract] OR Oman[Title/Abstract] OR Pakistan[Title/Abstract] OR Palau[Title/Abstract] OR Palestine[Title/Abstract] OR Panama[Title/Abstract] OR "Papua New Guinea"[Title/Abstract] OR Paraguay[Title/Abstract] OR Peru[Title/Abstract] OR Philippines[Title/Abstract] OR Poland[Title/Abstract] OR Portugal[Title/Abstract] OR Qatar[Title/Abstract] OR Romania[Title/Abstract] OR Russia[Title/Abstract] OR Rwanda[Title/Abstract] OR "Saint Kitts and Nevis"[Title/Abstract] OR "Saint Lucia"[Title/Abstract] OR "Saint Vincent and the Grenadines"[Title/Abstract] OR Samoa[Title/Abstract] OR "San Marino"[Title/Abstract] OR "Sao Tome and Principe"[Title/Abstract] OR "Saudi Arabia"[Title/Abstract] OR Senegal[Title/Abstract] OR Serbia[Title/Abstract] OR Seychelles[Title/Abstract] OR "Sierra Leone"[Title/Abstract] OR Singapore[Title/Abstract] OR Slovakia[Title/Abstract] OR Slovenia[Title/Abstract] OR "Solomon Islands"[Title/Abstract] OR Somalia[Title/Abstract] OR "South Africa"[Title/Abstract] OR "South Korea"[Title/Abstract] OR "South Sudan"[Title/Abstract] OR Spain[Title/Abstract] OR "Sri Lanka"[Title/Abstract] OR Sudan[Title/Abstract] OR Suriname[Title/Abstract] OR Sweden[Title/Abstract] OR Switzerland[Title/Abstract] OR Syria[Title/Abstract] OR Tajikistan[Title/Abstract] OR Tanzania[Title/Abstract] OR Thailand[Title/Abstract] OR Timor-Leste[Title/Abstract] OR Togo[Title/Abstract] OR Tonga[Title/Abstract] OR "Trinidad and Tobago"[Title/Abstract] OR Tunisia[Title/Abstract] OR Turkey[Title/Abstract] OR Turkmenistan[Title/Abstract] OR Tuvalu[Title/Abstract] OR Uganda[Title/Abstract] OR Ukraine[Title/Abstract] OR "United Arab Emirates"[Title/Abstract] OR "United Kingdom"[Title/Abstract] OR "United States of America"[Title/Abstract] OR Uruguay[Title/Abstract] OR Uzbekistan[Title/Abstract] OR Vanuatu[Title/Abstract] OR Venezuela[Title/Abstract] OR Vietnam[Title/Abstract] OR Yemen[Title/Abstract] OR Zambia[Title/Abstract] OR Zimbabwe[Title/Abstract]) AND AGREE[Title/Abstract] AND quality[Title/Abstract]

2. Methods – Qualitative study

The focus groups were conducted by DS, Phd, MD (male), who at the time was an assistant professor at the Center for Evidence-Based Medicine and Health Care, Catholic University of Croatia, Zagreb, Croatia. In his previous studies, DS has conducted several focus groups [3,4] and is a recognized expert in the field of qualitative analysis.

The moderator did not have any relationship with the participants prior to the focus groups, other than a very superficial acquaintance, which is to be expected in a small country like Croatia. Consequently, the participants had no knowledge about the moderator. Apart from DS's basic characteristics disclosed in this document, no other characteristics are reported in the main text.

We used purposive sampling with the method of approach including face-to-face, telephone, and/or email. We enrolled 11 participants in total, assigned to the two focus groups. Potential participants were reluctant to enter the study due to their busy schedules, but we did not keep records of the response rate.

At the start of the focus group (only the participants and the moderator were present), the moderator briefly introduced the participants to the project's goal. While the participants primarily led the general conversation, the welcome and closure of the session were scripted to convey the needed introduction, ground rules, and information about recordings in the most time effective manner [5]. We also scripted the first question cited in the main text of this study. Participants were informed during the introduction that the sessions would be audio recorded for transcription reasons. No field notes were taken and there were no repeat interviews. As time passed between interviews and analysis, we did not expect that returning transcripts to participants would provide reliable information so we did not them returned to participants.

TK performed initial coding that was then supervised by AJ. While the number of participants per group was adequate to identify majority of themes [6], due to reluctant enrolment of developers and users in the study, we conducted two focus groups. Nonetheless, Guest et al. demonstrated that more than 80% of themes in qualitative studies can be identified in 2 to 3 focus groups.

Participants did not provide feedback on the findings.

3. Supplementary figures and tables

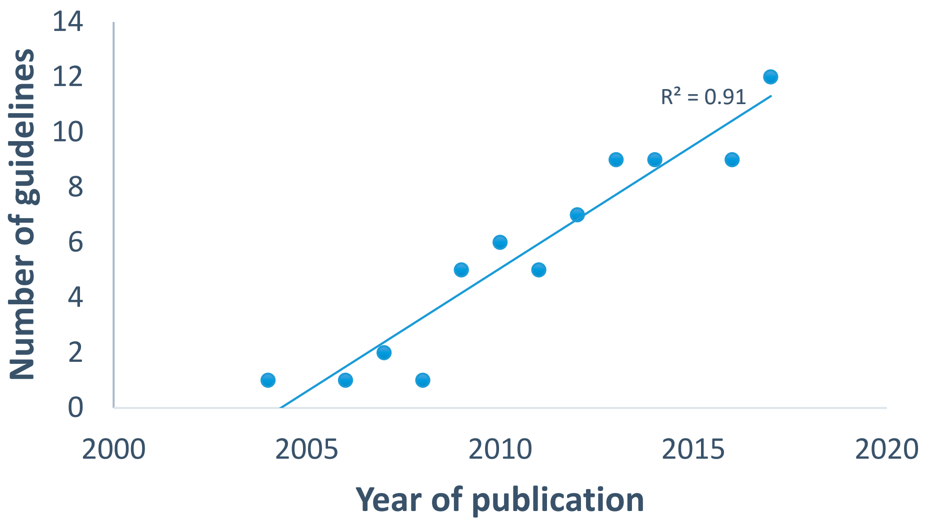

Supplementary Figure S1. The increase in the number of published clinical practice guidelines in Croatia up to 2017 (R^2=91%, $P_{regression\_coefficient}$<0.001)

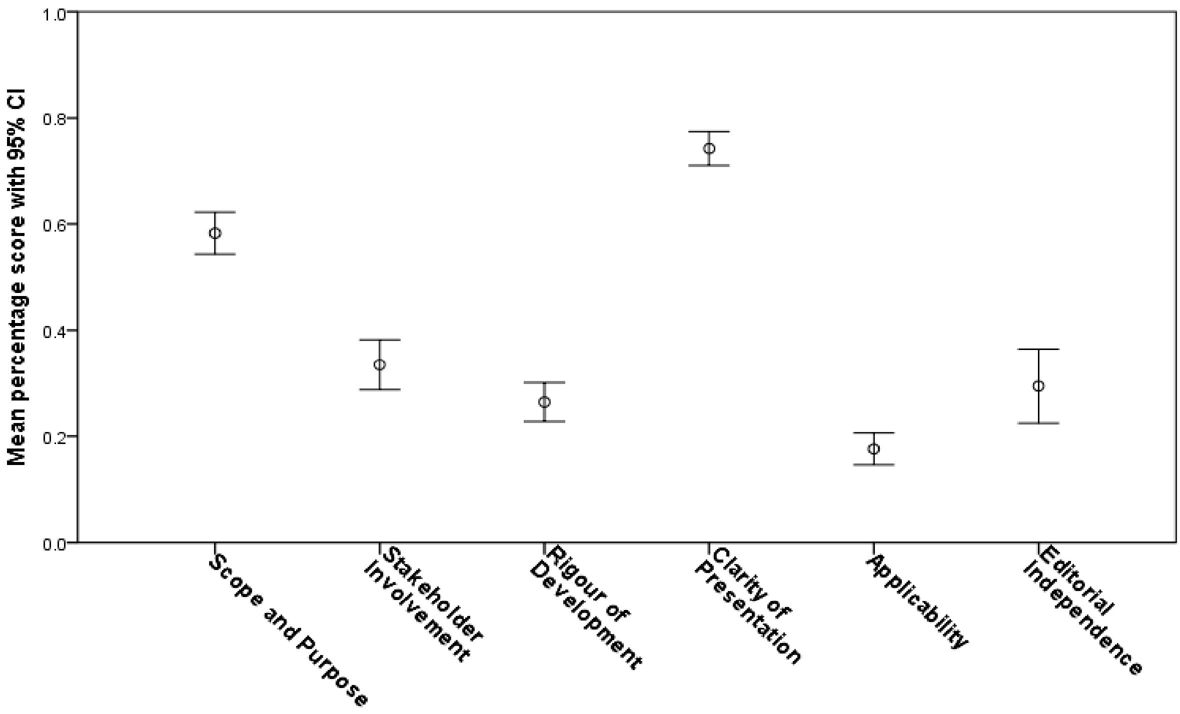

Supplementary Figure S2. AGREE II domain scores as a mean percentage of the maximum score with 95% confidence interval (95% CI). Domains with non-overlapping CIs present with significantly different scores.

Supplementary Table S1. The distribution of overall assessment AGREE II scores (7-point scale) of Croatian CPGs.

Overall Assessment (score 1-7; 1 – strongly disagree, and 7 – strongly agree.)	Frequency	Cumulative percentage
1	0	0%
2	7	10%
3	23	41%
4	26	76%
5	13	93%
6	5	100%
7	0	100%

Supplementary Table S2. Guideline frameworks in other countries and overall quality of the national guidelines

Country	Framework - guidelines developed by	Guidelines published for the years	Overall quality*	Reference No
Mexico	The National Healthcare Technology Excellence Center (acronym in Spanish: CENETEC). CENETEC is a governmental agency that was founded in 2004 and includes all the governmental Mexican Healthcare institutions in order to produce all the national CPGs.	2015-2017	Low	[7]
Phylipines	Predominantly local medical societies.	1995-2016	Low	[8]
Chile	The Disease Prevention and Control Division of the Ministry of Health.	2005-2016	Low	[9]
Argentina	Predominantly scientific societies.	1994-2004	Low	[10]
Japan	The Ministry of Health, Labor, and Welfare in Japan has pushed academic societies to develop guidelines, which are now developed and managed by academic societies and research groups. In addition, several other bodies/organizations maintain a clearinghouse for guidelines.	2004-2014	High	[11]
Australia	Various national initiatives in Australia exist to increase the accessibility and rigor of guidelines. For example, the National Health and Medical Research Council's repository of guidelines, founded in 2010, contain both NHMRC approved and non-certified CPGs and is designed to be a one-stop-shop for Australian CPGs.	2011-2018	High	[12]

***as cited in the original paper**

4. References

1. Brouwers MC, Makarski J, Durocher LD and Levinson A. E-learning interventions are comparable to user's manual in a randomized trial of training strategies for the AGREE II. *Implement Sci* 2011, 6:81, doi:10.1186/1748-5908-6-81.

2. Attal N, Cruccu G, Haanpää M, Hansson P, Jensen TS, Nurmikko T, et al. EFNS guidelines on pharmacological treatment of neuropathic pain. *Eur J Neurol* 2006, 13:1153–1169, doi:10.1111/j.1468-1331.2006.01511.x.

3. Marinovic D, Hren D, Sambunjak D, Rašić I, Škegro I, Marušić A, et al. Transition from longitudinal to block structure of preclinical courses: outcomes and experiences. *Croat Med J* 2009, 50(5):492-506, doi:10.3325/cmj.2009.50.492.

4. Bloom JD, Hoxha I, Sambunjak D, Sondorp E. Ethnic segregation in Kosovo's post-war health care system. *Eur J Public Health* 2007, 17(5):430-436, doi:10.1093/eurpub/ckl270.

5. Then KL, Rankin JA, Ali E. Focus group research: what is it and how can it be used? *Can J Cardiovasc Nurs* 2014, 24(1):16-22.

6. Guest G, Namey E and McKenna K. How many focus groups are enough? Building an evidence base for nonprobability sample sizes. *Field Methods* 2016, 29(1):3-22, https://doi.org/10.1177/1525822X16639015.

7. Colunga-Lozano LE, Gerardo-Morales V, Perez-Gaxiola G, Vazquez-Alvarez AO, Gonzalez-Torres FJ, Perales-Guerrero L, et al. Methodological assessment of Mexican clinical practice guidelines: GRADE framework adherence and critical appraisal. *J Eval Clin Pract* 2021, 27(2):385-390, doi:10.1111/jep.13447.

8. Dans LF, Salaveria-Imperial MLA, Miguel RTD, Tan-Lim CSC, Eubanas GAS, Tolosa MTS, et al. Guidelines in low and middle income countries Paper 3: appraisal of Philippine clinical practice guidelines using Appraisal of guidelines for research and evaluation II: improvement needed for rigor, applicability, and editorial independence. *J Clin Epidemiol* 2020, 127:184-190, doi:10.1016/j.jclinepi.2020.06.036.

9. Loezar C, Perez-Bracchiglione J, Arancibia M, Meza N, Vargas M, Papuzinski C, et al. Guidelines in low and middle income countries Paper 2: Quality assessment of Chilean guidelines: need for improvement in rigor, applicability, updating, and patients' inclusion. *J Clin Epidemiol* 2020, 127:177-183, doi:10.1016/j.jclinepi.2020.07.018.

10. Esandi ME, Ortiz Z, Chapman E, Dieguez MG, Mejia R, Bernztein R. Production and quality of clinical practice guidelines in Argentina (1994-2004): a cross-sectional study. *Implement Sci* 2008, 3:43, doi:10.1186/1748-5908-3-43.

11. Seto K, Matsumoto K, Fujita S, Kitazawa T, Amin R, Hatakeyama Y, et al. Quality assessment of clinical practice guidelines using the AGREE instrument in Japan: a time trend analysis. *PloS one* 2019, 14(5):e0216346, doi:10.1371/journal.pone.0216346.

12. Barker TH, Dias M, Stern C, Porritt K, Wiechula R, Aromataris E, et al. Guidelines rarely used GRADE and applied methods inconsistently: a methodological study of Australian guidelines. *J Clin Epidemiol* 2020, 130:125-134, doi:10.1016/j.jclinepi.2020.10.017.
